# A systematic review of biomarkers in Takotsubo syndrome: A focus on better understanding the pathophysiology

**DOI:** 10.1016/j.ijcha.2021.100795

**Published:** 2021-05-19

**Authors:** Hilal Khan, David Gamble, Alice Mezincescu, Hassan Abbas, Amelia Rudd, Dana Dawson

**Affiliations:** Cardiology Research Group, Aberdeen Cardiovascular and Diabetes Centre, School of Medicine and Dentistry, University of Aberdeen, Foresterhill Aberdeen AB25 2ZD, United Kingdom

**Keywords:** Takotsubo Syndrome, Biomarkers, Pathophysiology, Diagnosis, Prognosis, Systematic review

## Abstract

**Background:**

The diagnosis of Takotsubo syndrome is made based on clinical presentation, ECG, biomarker, imaging and coronary angiography. There is a lack of diagnostic biomarkers that can discriminate patients with Takotsubo syndrome from those with acute myocardial infarction (AMI) and provide clinical monitoring and prognostic information in the long-term.

**Methods:**

A literature search of published Takotsubo syndrome biomarkers from PubMed was performed. All studies that included numerical biomarker data on Takotsubo syndrome was included. Exclusion criteria was any study without an AMI cohort for comparison in the acute phase biomarkers or due to the absence of numerical values. The results were tabulated in table form with results expressed as either mean ± SD or median (interquartile range).

**Results:**

The literature search produced 14 relevant studies that met search criteria**.** The results showed; high sensitivity Troponin I (3.21 ± 4.4 vs 34.4 ± 37 ng/ml), BNP [972 (578.5–1671.0) pg/L vs 358 (50.5–688.0) pg/L in NSTEMI and vs 381 (106.0–934.0) pg/L in STEMI] and BNP/Troponin I ratio [642 (331.8–1226.5) vs 184.5 (50.5–372.3) pg/ug in NSTEMI and 7.5 (2.0–29.6) pg/ug in STEMI] patients.

**Discussion:**

This study is limited by many studies being retrospective cohort studies. This data shows that acutely troponin is raised in Takotsubo syndrome but not enough to be discriminating from AMI. BNP level is significantly raised in Takotsubo syndrome compared to AMI.

**Conclusion:**

Current specificity of acute and chronic biomarkers for Takotsubo syndrome is lacking and further work is needed to address the gap in knowledge.

## Highlights

• Biomarkers play an important role in our understanding of Takotsubo syndrome.
• Several biomarkers of interest exist in Takotsubo syndrome.
• The BNP/Troponin ratio appears to have the most promise from a diagnostic standpoint.
• There is a large inflammatory cascade in Takotsubo syndrome.

## 1 Introduction

Takotsubo syndrome (stress induced cardiomyopathy), is a recently described acute cardiac presentation that mimics an acute myocardial infarction (AMI). The current diagnostic criteria for Takotsubo typically focus on AMI-like symptoms and ECG changes, cardiac biomarker release, imaging evidence of left ventricular dysfunction – often of transient nature - and intense myocardial oedema, in the absence of causative culprit plaque on coronary angiography [1], [2].

Patients with Takotsubo syndrome usually present with chest pain and ECG changes which are unable to reliably differentiate them from patients with AMI. For those presenting with ST-elevation the diagnosis is rapidly established with invasive coronary angiography. A diagnostic biomarker, no matter how specific is unlikely to be of additional utility or to replace the need for coronary angiography in this group of patients. The majority of Takotsubo patients present with non-ST elevation and undergo in-hospital stay and pre-coronary angiography treatment typical for AMI so they are commonly prescribed antiplatelet and anti-coagulant therapy, for which there is no evidence of clinical benefit [3]. In such patients a diagnostic biomarker could be utilised in conjunction with non-invasive coronary angiography and the already described differentiating features of the ECG evolution over the initial 3 days, specifically deep and widespread T wave inversion and increased QTc interval [4]. In addition, cardiac magnetic resonance imaging will exclude fibrosis (shown as late gadolinium enhancement) and confirm the presence of myocardial oedema which usually confirms the diagnosis [5].

Whilst a *specific* diagnostic biomarker for Takotsubo syndrome does not exist and may be difficult to envisage before addressing the causative pathophysiology of the disease, matters may be different regarding the clinical utility of a biomarker used in recovery, convalescence, or long-term follow-up. From a prognostic and therapeutic perspective, biomarker monitoring may help select the patient population who may benefit from medical therapies (such as ACE-inhibition(1) suggested by registry data) or guide the duration of such treatments, given the dynamic nature of the condition (for example first 3–6 months versus life-long). In addition, a pure understanding of the biomarker profile in Takotsubo syndrome may allow a better understanding of the pathophysiology of the condition which will aid in the development of better treatments in the future.

Here, we review the literature available on biomarkers in Takotsubo syndrome. From an acute perspective we focused on a comparison with patients with AMI to identify clinically relevant differences that may guide clinicians in early detection, investigation and management of acute Takotsubo syndrome. From a chronic perspective we focus on biomarkers with a role in disease monitoring and risk stratification, we visit several pathophysiological pathways that may be important in understanding the mechanism behind Takotsubo syndrome.

## 2 Methods

### 2.1 Study design

The study was designed as a systematic review (see Table 3, Table 4, Table 5). A literature search of published Takotsubo syndrome biomarkers (1990 to 2021, English language only) from PubMed Library was performed. Search terms included: (biomarkers in Takotsubo cardiomyopathy), (biomarkers in Takotsubo syndrome), (markers of mortality in Takotsubo cardiomyopathy), (biomarkers in stress induced cardiomyopathy), (markers of death in Takotsubo cardiomyopathy), (markers of mortality in stress induced cardiomyopathy), (markers of death in stress induced cardiomyopathy). Relevant mean/median values, standard deviations, or standard errors of the mean and/or range were noted, if available, as well as timing of sampling relative to admission.

### 2.2 Eligibility criteria

We included adult patients (18 years and older) with a diagnosis of Takotsubo syndrome based either on the Mayo clinic criteria or the European society of cardiology criteria. All studies included had performed repeat imaging after several months to confirm the diagnosis of Takotsubo syndrome.

### 2.3 Exclusion criteria

We excluded any studies which did not include an AMI comparator group when assessing acute biomarkers. We excluded studies which did not have any control group for chronic biomarkers. In addition, we excluded case reports of biomarkers in Takotsubo syndrome. We also excluded studies which did not present biomarker data in numerical values with a mean ± SD or median (interquartile range).

### 2.4 Data items and data collection

We collected data on the author of the study, the year of the study, the number of patients included in the study, the unit of measurement of the biomarker in question, timing of sample from admission, the time from symptom onset to admission, the value of the biomarker in Takotsubo syndrome, the value of the biomarker in AMI patients, the p-value and the normal reference range if available.

## 3 Results

### 3.1 Literature selection

**Fig. 1:**
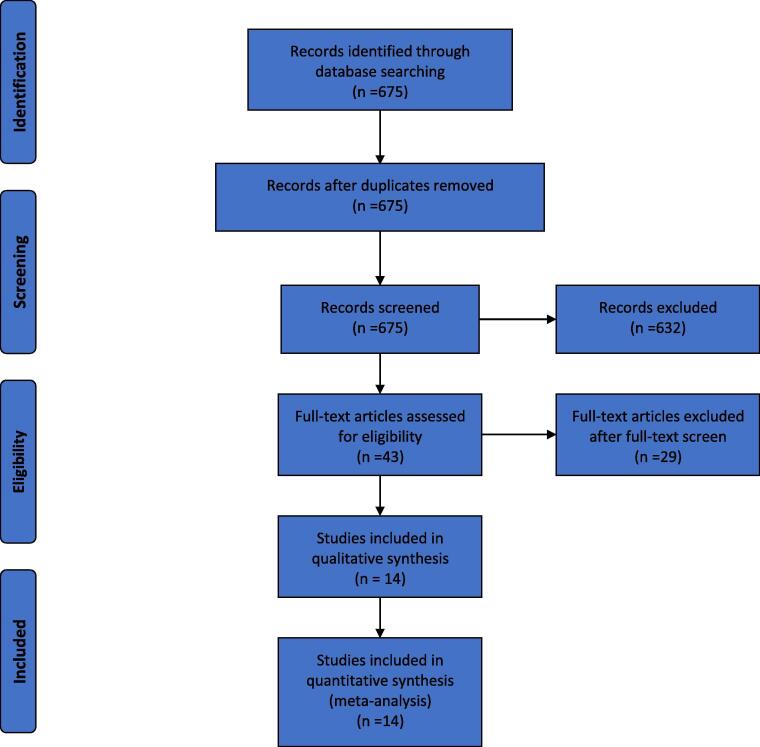
Systematic literature review search flow diagram.

The initial search strategy produced 675 articles. After screening the article abstract the number reduced to 43 articles. A further 29 papers were removed after reading of the full text due to an absence of either an AMI cohort for comparison in the acute phase biomarkers or due to the absence of numerical values (results presented only in graphical format). Fourteen papers were included in the final data analysis (Fig. 1). All these studies used the Mayo clinic criteria to diagnose patients with Takotsubo syndrome.

### 3.2 Biomarkers in acute Takotsubo syndrome

We identified 13 papers that reported one or several acute biomarkers in patients with Takotsubo syndrome compared to patients with AMI, reporting on a total of 1194 patients.

Table 1 presents 27 biomarkers investigated during the acute phase. These were subdivided into 5 categories:

**Table 1:** Biomarkers in acute Takotsubo syndrome.

a.	b. Markers of cardiac injury/stretch								
Author	Time from symptom onset	Year	No patients	Biomarker (units)	Timing of sample	Takotsubo syndrome	Acute Myocardial Infarction	P-Value	Reference range-Normal
	*Troponin I*								
Doyen et al. [6]	NA	2014	152	ng/mL	TnI^a^ at peak^b^	1.6 (0.7–3.1)^α^	2.1 (0.4–9.4) (NSTEMI)^c^ 51.4 (27.6–80.1) (STEMI)^d^	0.319 < 0.001	<0.06
Santoro et al. [12]	NA	2018	64	ng/ml	TnI at admission	3.21 ± 4.4^β^	34.4 ± 37 (ACS)^e^	0.01	<0.5
Nascimento et al. [35]	NA	2012	154	ng/dl	TnI at 8–12 h	7.6 ± 18	102.2 +/- 110.3 (STEMI)	<0.001	NA
Budnik et al. [11]	TTS-8 h AMI-5 h	2015	132	ng/ml	TnI at 12 h	2.1 (0.7–4.0)	19 (7.4–52.9) (STEMI)	< 0.001	NA

	*Troponin T*								
Randhawa et al. [9]	NA	2014	155	ng/ml	Troponin T at admission	0.38 (0.16–0.65)	0.52 (0.19–1.45) (AMI)^f^	0.0092	NA
Frohlich et al. [10]	TTS-7.5 h STEMI-6 h NSTEMI-10 h	2012	121	ug/l	Troponin T at admission	0.34 (0.15–0.61)	1.02 (0.40–3.37) (NSTEMI) 2.55 (0.35–6.91) (STEMI)	<0.001	NA

	*Creatine Phosphokinase*								
Doyen et al. [6]	NA	2014	152	UI/L	CPK^g^ at peak^b^	193 (98–308)	199 (99–390) (NSTEMI) 1501 (1087–2851) (STEMI)	0.476 < 0.001	NA
Frohlich et al. [10]	TTS-7.5 h STEMI-6 h NSTEMI-10 h	2012	121	U/L	CPK on day 1	145 (93–239)	1364 (615–1751) (NSTEMI) 2006 (1251–3951) (STEMI)	< 0.001 < 0.001	NA

	*Creatine Phosphokinase MB fraction*								
Doyen et al. [6]	NA	2014	152	UI/L	CPK-MB^h^ at peak^b^	37 (27–46)	39 (27–67) (NSTEMI) 211 (115–407) (STEMI)	0.171 < 0.001	NA
Randhawa et al. [9]	NA	2014	155	ng/ml	CPK-MB at admission	10.5 (5.8–14.9)	25.0 (13.8–62.7) (AMI)	<0.0001	NA
Frohlich et al. [10]	TTS-7.5 h STEMI-6 h NSTEMI-10 h	2012	121	U/l	CPK-MB on day 1	36 (22–48)	137 (71–242) (NSTEMI) 258 (107–395) (STEMI)	< 0.001 < 0.001	NA
Budnik et al. [11]	TTS-8 h AMI-5 h	2015	132	ng/ml	CPK-MB at 12 h	9.5 (3.2–21.3)	73.3 (26.8–151.7) (STEMI)	< 0.001	NA

	*Myoglobin*								
Doyen et al. [6]	NA	2014	152	UI/L	Myoglobin at peak^b^	69.5 (50.5–152.5)	81 (35.5–149.0) (NSTEMI) 331 (188.0–637.0) (STEMI)	0.986 <0.001	NA
Frohlich et al. [10]	TTS-7.5 h STEMI-6 h NSTEMI-10 h	2012	121	ug/l	Myoglobin at admission	85.0 (46.0–167)	506 (134–1032) (NSTEMI) 729 (359–2234) (STEMI)	< 0.001 < 0.001	NA

	*Brain natriuretic peptide*								
Doyen et al. [6]	NA	2014	152	pg/L	BNP^i^ at admission	972 (578.5–1671.0)	358 (50.5–688.0) (NSTEMI) 381 (106.0–934.0) (STEMI)	<0.001 <0.001	<100
Randhawa et al. [9]	NA	2014	155	pg/ml	BNP at admission	456.5 (120.25–734.5)	97 (45.5–248.5)	<0.0001	NA
Frohlich et al. [10]	TTS-7.5 h STEMI-6 h NSTEMI-10 h	2012	121	ng/L	NT-pro-BNP^j^ at admission	1723 (754–5699)	977 (499–2290) (NSTEMI) 461 (188–1451) (STEMI)	0.001 0.001	NA
Budnik et al. [11]	TTS-8 h AMI-5 h	2015	132	ng/ml	NT-pro-BNP at 12 h	4702 (2530–8350)	2138 (1013–4647) (STEMI)	0.002	NA

	*BNP/Tn I ratio*								
Doyen et al. [6]	NA	2014	152		BNP at admission Tn I-peak^b^	642 (331.8–1226.5)	184.5 (50.5–372.3) (NSTEMI) 7.5 (2.0–29.6) (STEMI)	<0.001 <0.001	NA
Budnik et al. [11]	TTS-8 h AMI-5 h	2015	132		NT-pro-BNP and Tn-I at 12 h	2235.2 (1086.2–9480.8)	81.6 (47.9–383.3) (STEMI)	< 0.001	NA

	*hs-TnT/CKMB^k^*								
Pirlet et al. [36]	TTS-20 h NSTEMI-7.7 h STEMI-3.5 h	2017	83		Hs-TnT and CKMB at admission Hs-TnT and CKMB at peak^b^	0.024 (0.018–0.047) 0.029 (0.023–0.045)	0.009 (0.006–0.022) (NSTEMI) 0.011 (0.006–0.016) (STEMI) 0.018 (0.011–0.033) (NSTEMI) 0.011 (0.007–0.016) (STEMI)	<0.0001 0.0002 0.002 <0.001	NA

	*BNP/TnT*								
Randhawa et al. [9]	NA	2014	155		BNP and TnT at admission	1,292.1 (443.4–2,657.9)	226.9 [69.91–426.32] (AMI)	<0.001	NA

	*BNP/CKMB*								
Randhawa et al. [9]	NA	2014	155		BNP and CKMB at admission	28.4 (13.7–94.8)	3.6 (1.1–10.0) (AMI)	<0.001	NA

c.	d. Markers of the Immune/Inflammatory response								
Author	Time from symptom onset	Year	No patients	Biomarker (units)	Timing of sample	Takotsubo Syndrome	Acute Myocardial Infarction	p value	Reference range - normal
	*Interleukin-1β*								
Santoro et al. [12]	NA	2018	64	pg/ml	IL-1β^l^ at 0 h IL-1β at 2 h	1.50 ± 1.54 1.96 ± 2.01	1.25 ±1.24 (ACS) 0.87 ±1.23 (ACS)	0.47 0.01	0.5–1.6

	*Interleukin-6*								
Santoro et al. [12]	NA	2018	64	pg/ml	IL-6^m^ at 0 h IL-6 at 2 h	112.4 ± 17.5 7.4 ± 7.1	25.4 ± 27.7 (ACS) 19.6 ± 23.1 (ACS)	0.03 0.02	1.2–2.0
Pirzer et al. [14]	TTS-6 h AMI-6 h	2012	32	pg/ml	IL-6 at admission	2.1 ± 2.6	5.2 ± 5.0 (ACS)		NA

	*Interferon-γ*								
Santoro et al. [12]	NA	2018	64	pg/ml	IFN-γ^n^ at 0 h IFN-γ at 2 h	0.92 ± 0.64 1.45 ± 1.41	0.32 ± 0.47 (ACS) 0.41 ± 0.68 (ACS)	0.02 0.01	0.34–0.50

	*Tissue necrosis factor-α*								
Santoro et al. [12]	NA	2018	64	pg/ml	TNF-α^o^ at 0 h TNF-α at 2 h	5.0 ± 4.7 4.44 ± 2.88	2.3 ± 2.0 (ACS) 3.09 ± 3.90 (ACS)	0.01 0.09	0.5–5.0

	*Interleukin-2*								
Santoro et al. [12]	NA	2018	64	pg/ml	IL-2^p^ at 0 h IL-2 at 2 h	2.0 ± 1.5 4.6 ± 5.3	0.5 ± 0.1 (ACS) 0.7 ± 1.2 (ACS)	0.01 0.01	4.8–8.7

	*Interleukin-4*								
Santoro et al. [12]	NA	2018	64	pg/ml	IL-4^q^ at 0 h IL-4 at 2 h	1.5 ± 1.0 1.6 ± 0.7	0.8 ± 1.1 (ACS) 0.8 ± 1.5 (ACS)	0.02 0.09	0.5–6.6

	*Interleukin-10*								
Santoro et al. [12]	NA	2018	64	pg/ml	IL-10^r^ at 0 h IL-10 at 2 h	3.3 ± 3.8 2.8 ± 3.5	1.6 ± 2.2 (ACS) 1.4 ± 3.5 (ACS)	0.03 0.06	0.1–1.8

	*Suppression-of-tumorigenicity 2*								
Hojagergaard et al. [23]	NA	2019	60	ng/ml	Suppression-of-tumorigenicity 2 at admission	53 (32–157)	45 (31–55) (STEMI)	0.008	NA

	*Soluble Thrombomodulin*								
Hojagergaard et al. [23]	NA	2019	60	ng/ml	Soluble Thrombomodulin at admission	7.9 (5.9–9.6)	6.4 (5.5–7.8) (STEMI)	0.04	NA

e.	f. Growth factor markers								
Author	Time from symptom onset	Year	No patients	Biomarker (units)	Timing of sample	Takotsubo Syndrome	Acute Myocardial Infarction	p value	Reference range - Normal
	*Growth differentiation factor-15*								
Stiermaier et al. [37]	NA	2011	44	ng/l	Growth differentiation factor-15 at admission	3047 (2256–7572)	1527 (1152–2677) (STEMI)	0.002	<1200

	*Endothelial growth factor*								
Santoro et al. [12]	NA	2018	64	pg/ml	EGF^s^ at 0 h EGF at 2 h	84.8 ± 42.9 36.3 ± 18.5	10.7 ± 11.2 (ACS) 18.5 ± 25.3 (ACS)	0.01 0.03	2.9–271

g.	h. Markers of Vascular Stress								
Author	Time from symptom onset	Year	No patients	Biomarker (units)	Timing of sample	Takotsubo Syndrome	Acute Myocardial Infarction	p value	Reference range - Normal
	*Copeptin*								
Hojagergaard et al. [23]	NA	2019	60	pmol/l	Copeptin at admission	10.4 (7.6–39.0)	92.3 (13–197) (STEMI)	0.008	NA
Budnik et al. [24]	TTS-12 h AMI-12 h	2020	29	ng/ml	Copeptin at admission	0.49 (0.45–1.21)	1.55 (1.34 – 1.65) (STEMI)	0.002	NA

	*Glycocalyx*								
Nguyen et al. [19]	NA	2016	77	Syndecan-1 μg/L	Syndecan-1 at admission	97 ± 65 41 ± 10 (healthy controls)	256 ± 208 (AMI)	< 0.0001 0.005	NA

i.	j. Messenger markers								
Author	Time from symptom onset	Year	No patients	Biomarker (units)	Timing of sample	Takotsubo Syndrome	Acute Myocardial Infarction	p value	Reference range - Normal
	*MicroRNA*								
Jaguszewski et al. [22]	TTS-24 h AMI-24 h	2013	91	miR-16 (AUC)	miR-16^t^ at admission	0.76 (0.64 – 0.88) ^γ^	0.78 (0.66 – 0.89)^δ^	<0.001	NA
Jaguszewski et al. [22]	TTS-24 h AMI-24 h	2013	91	miR-26a (AUC)	miR-26a^u^ at admission	0.73 (0.59 – 0.86) ^γ^	0.70 (0.56 –0.84)^δ^	<0.01	NA
Jaguszewski et al. [22]	TTS-24 h AMI-24 h	2013	91	miR-133a (AUC)	miR-133a^v^ at admission	0.76 (0.63–0.88) ^γ^	0.75 (0.62–0.89)^δ^	<0.001	NA
Jaguszewski et al. [22]	TTS-24 h AMI-24 h	2013	91	miR-1 (AUC)^1^	miR-1^w^ at admission	0.78 (0.66–0.90)^3^	0.64 (0.50–0.79)^4^	<0.001^3^ 0.06^4^	NA

TnI- Troponin I α) Value represented as median [interquartile range].

Levels measured every 6 h, peak defined as maximal level before decrease in biomarker β) Value represented as mean ± standard deviation.

NSTEMI – Non-ST elevation myocardial infarction γ) Area Under Curve – Takotsubo Syndrome versus Healthy Controls.

STEMI – ST elevation myocardial infarction δ) Area Under Curve – Takotsubo Syndrome versus STEMI.

ACS – Acute Coronary Syndrome TTS = takostubo syndrome, AMI = acute myocardial infarction, NA = not available.

AMI – Acute Myocardial Infarction.

CPK-Creatinine phosphokinase.

CPK-MB-Creatinine phosphokinase MB isoform.

BNP-Brain natriuretic peptide.

NT-pro-BNP- N terminal - pro-Brain natriuretic peptide.

hs-TnT/CKMB- high sensitivity troponin T/Creatinine kinase MB.

IL-1β - Interleukin-1β.

IL-6- Interleukin-6.

IFN-γ- Interferon- γ.

TNF-α- Tissue necrosis factor- α.

IL-2- Interleukin-2.

IL-4-Interleukin-4.

IL-10-Interleukin-10.

EGF- endothelial growth factor.

miR16- MicroRNA 16.

miR26a- MicroRNA 26a.

miR133a- MicroRNA 133a.

miR-1- MicroRNA 1.

#### 3.2.1 Markers of cardiac injury/stretch

Table 1a shows the classical markers of cardiac injury - Troponin I, Troponin T (TnT), Creatine Phosphokinase (CK), Creatine Phosphokinase MB fraction (CKMB), Myoglobin - and stretch – BNP - as well as various ratios between them: BNP/Troponin I ratio, hs-TnT/CKMB, BNP/TnT and BNP/CKMB.

The peak Troponin I ranged between 1.6 (0.7–3.1) ng/mL, 7.6 ng/dl ± 18, 2.1 ng/ml (0.7–4.0) in TTC compared to 51.4 (27.6–80.1) ng/mL, 19 ng/ml (7.4–52.9), 102.2 ng/dl ± 110.3 in STEMI patients. The brain natriuretic peptide (BNP) level at admission in patients with TTC is 972 (578.5–1671.0) pg/L, 456.5 pg/ml [120.25–734.5] compared to 358 (50.5–688.0) pg/L in NSTEMI, 381 (106.0–934.0) pg/L in STEMI. The BNP/TnI ratio is 642 (331.8–1226.5) pg/ug in Takotsubo cardiomyopathy and 184.5 (50.5–372.3) pg/ug in NSTEMI and 7.5 (2.0–29.6) pg/ug in STEMI patients.

#### 3.2.2 Markers of the Immune/Inflammatory response

Table 1b elaborates the pro- and anti-inflammatory cytokines produced by activation of the innate immune system such as phagocytic leucocytes or activated immune tissue cells such as macrophages and dendritic cells: the interleukins family (IL-1β, IL-6, IFN-γ, TNF-α, IL-2, IL-4, IL-10), Suppression-of-tumorigenicity 2 and Soluble Thrombomodulin.

The IL-6 level is 112.37 pg/ml ± 17.48 at 0 h and 7.35 pg/ml ± 7.10 at 2 h in TTC compared to 25.42 pg/ml ± 27.71 at 0 h and 19.6 pg/ml ± 23.05 at 2 h in AMI patients, Pirzer et al showed that IL-6 levels at admission in TTC patients is 2.1 pg/ml ± 2.6 compared to 5.2 pg/ml +/- 5.0 in patients with AMI. The Interferon (IFN)-γ level is 0.92 pg/ml ± 0.64 at 0 h and 1.45 pg/ml ± 1.41 at 2 h in TTC compared to 0.32 pg/ml ± 0.47 at 0 h and 0.41 pg/ml ± 0.68 at 2 h in AMI patients. The Tissue necrosis factor (TNF)-α level is 5.02 pg/ml ± 4.7 at 0 h and 4.44 pg/ml ± 2.88 at 2 h in TTC compared to 2.33 pg/ml ± 1.98 at 0 h and 3.09 pg/ml ± 3.90 at 2 h in AMI patients

#### 3.2.3 Growth factor markers (promoting proliferation, apoptosis or differentiation)

Table 1c shows the activation of Growth differentiation factor-15 and endothelial growth factor (EGF) – as signalling molecules promoting repair and differentiation.

The Growth differentiation factor 15 (GDF-15) levels at admission were between 3047 ng/l (2256–7572) ng/l in TTC patients compared to 1527 ng/l (1152–2677) in STEMI patients. The Endothelial growth factor (EGF) level is 84.77 pg/ml ± 42.91 at 0 h and 36.32 pg/ml ± 18.46 at 2 h in TTC compared to 10.65 pg/ml ± 11.22 at 0 h and 18.49 pg/ml ± 25.33 at 2 h in AMI patients.

#### 3.2.4 Markers of vascular stress (haemodynamic, ischemic, metabolic)

Table 1d shows the information derived from the contribution of vascular responses in Takotsubo syndrome, the most studied are Copeptin (peptide derived from the C-terminus of pre-pro-hormone arginine vasopressin) and Syndecan-1 (a *trans*-membrane proteoglycan) as biomarkers of vascular stress.

The copeptin level is 10.4 pmol/l (7.6–39) and 0.49 ng/ml (0.45–1.21) at admission in TTC patients compared to 92.3 pmol/l (13–197) and 1.55 ng/ml (1.34–1.65) in STEMI patients.

#### 3.2.5 Circulating micro-RNA (miRNA) profiling of dysregulated pathways

Table 1e details families of circulating non-coding RNA molecules which function as post-transcriptional regulators of gene expression most recently recognised as biological regulators (miR-16, miR-26a, miR-133a, miR-1)**.**

The level of miR-16 is 0.76 (0.64 – 0.88) in Takotsubo syndrome and 0.78 (0.66 – 0.89) in AMI patients. The level of miR-26a is 0.73 (0.59 – 0.86) in Takotsubo syndrome and 0.70 (0.56 –0.84) in AMI.

### 3.3 Biomarkers in convalescent and chronic post-Takotsubo syndrome Stages

We identified 1 study that explored biomarkers in Takotsubo syndrome patients after the acute presentation. These were Troponin I, IL-6, IL-8, BNP studied in a total of 106 patients which are shown in Table 2.

**Table 2:** Biomarkers in Convalescent and Chronic Takotsubo syndrome Stages.

Chronic biomarkers in Takotsubo Syndrome								
Author	Year	No patients	Biomarker (units)	Timing of sample and cut-off	Takotsubo syndrome	Age and gender matched controls	P-Value	Reference range
*Troponin I*								
Scally et al. [2]	2019	106	(ng/L)	hsTroponin I^a^ at 5 months	6.47 ± 0.6*	NA	NA	<5 (detectable limit)

*Interleukin-6*								
Scally et al. [2]	2019	106	(pg/ml)	IL-6^b^ at 5 months	18.3 ± 5.17	6.5 ± 5.83	0.008	NA

*Interleukin-8*								
Scally et al. [2]	2019	106	(pg/ml)	IL-8^c^ at 5 months	61.9 ± 10.28	21.7 ± 10.86	0.009	NA

*BNP*^d^								
Scally et al. [2]	2019	106	(pg/ml)	BNP at 5 months	77.9 ± 45 (Mean ± SD)	32.7 ± 4.6	0.003	NA

a hsTroponin I- high sensitivity Troponin I.

b IL-6- Interleukin-6.

c IL-8- Interleukin-8.

d BNP- Brain natriuretic peptide.

* Value represented as mean ± standard deviation.

The hs TnI level in convalescent Takotsubo syndrome was 6.47 ± 0.6 (reference range < 5). The BNP level is 77.9 ± 45 in Takotsubo syndrome and 32.7 ± 4.6 in healthy controls. The IL-6 level is 18.3 ± 5.17 in Takotsubo syndrome and 6.5 ± 5.838 in healthy controls. The IL-8 level is 61.9 ± 10.28 in Takotsubo syndrome and 21.7 ± 10.86 in healthy controls.

**Table 3:** Assessment of the quality of studies included in the systematic review.

Study	Selection				Comparability	Outcome			Result
	Representativeness of the exposed cohort	Selection of the non exposed cohort	Ascertainment of exposure	Demonstration that outcome of interest was not present at start of study	Comparability of cohorts on the basis of the design or analysis	Assessment of outcome	Was follow-up long enough for outcomes to occur	Adequacy of follow up of cohorts	Score
Doyen et al. [6]	*	*	*	*	*	*	*	*	8
Randhawa et al. [9]	*	*	*	*	*	*	*	*	8
Frohlich et al. [10]	*	*	*	*	*	*	*	*	8
Budnik et al. [11]	*	*	*	*	*	*	*	*	8
Santoro et al. [12]	*	*	*	*	*	*	*	*	8
Pirzer et al. [14]	*	*	*	*	*	*	*	*	8
Nguyen et al. [19]	*	*	*	*	*	*	*	*	8
Jaguszewski et al. [22]	*	*	*	*	*	*	*	*	8
Hojagergaard et al. [23]	*	*	*	*	*	*	*	*	8
Budnik et al. [24]	*	*	*	*	*	*	*	*	8
Pirlet et al. [36]	*	*	*	*	*	*	*	*	8
Stiermaier et al. [37]	*	*	*	*	*	*	*	*	8

**Table 4:** PRISMA 2020 checklist.

Line 1Section and Topic	Item #	Checklist item	Location where item is reported
TITLE			
Title	1	Identify the report as a systematic review.	Line 1

ABSTRACT			
Abstract	2	See the PRISMA 2020 for Abstracts checklist.	See Abstract Checklist

INTRODUCTION			
Rationale	3	Describe the rationale for the review in the context of existing knowledge.	Line 66–95
Objectives	4	Provide an explicit statement of the objective(s) or question(s) the review addresses.	Line 97–102

METHODS			
Eligibility criteria	5	Specify the inclusion and exclusion criteria for the review and how studies were grouped for the syntheses.	Line 124–134
Information sources	6	Specify all databases, registers, websites, organisations, reference lists and other sources searched or consulted to identify studies. Specify the date when each source was last searched or consulted.	Line 114–123
Search strategy	7	Present the full search strategies for all databases, registers and websites, including any filters and limits used.	Line 115–121
Selection process	8	Specify the methods used to decide whether a study met the inclusion criteria of the review, including how many reviewers screened each record and each report retrieved, whether they worked independently, and if applicable, details of automation tools used in the process.	135–140
Data collection process	9	Specify the methods used to collect data from reports, including how many reviewers collected data from each report, whether they worked independently, any processes for obtaining or confirming data from study investigators, and if applicable, details of automation tools used in the process.	Line 135–140
Data items	10a	List and define all outcomes for which data were sought. Specify whether all results that were compatible with each outcome domain in each study were sought (e.g. for all measures, time points, analyses), and if not, the methods used to decide which results to collect.	Line 135–140
	10b	List and define all other variables for which data were sought (e.g. participant and intervention characteristics, funding sources). Describe any assumptions made about any missing or unclear information.	Line 135–140
Study risk of bias assessment	11	Specify the methods used to assess risk of bias in the included studies, including details of the tool(s) used, how many reviewers assessed each study and whether they worked independently, and if applicable, details of automation tools used in the process.	Table 3
Effect measures	12	Specify for each outcome the effect measure(s) (e.g. risk ratio, mean difference) used in the synthesis or presentation of results.	Line 121–123
Synthesis methods	13a	Describe the processes used to decide which studies were eligible for each synthesis (e.g. tabulating the study intervention characteristics and comparing against the planned groups for each synthesis (item #5)).	Line 124–140
	13b	Describe any methods required to prepare the data for presentation or synthesis, such as handling of missing summary statistics, or data conversions.	NA
	13c	Describe any methods used to tabulate or visually display results of individual studies and syntheses.	NA
	13d	Describe any methods used to synthesize results and provide a rationale for the choice(s). If *meta*-analysis was performed, describe the model(s), method(s) to identify the presence and extent of statistical heterogeneity, and software package(s) used.	NA
	13e	Describe any methods used to explore possible causes of heterogeneity among study results (e.g. subgroup analysis, *meta*-regression).	NA
	13f	Describe any sensitivity analyses conducted to assess robustness of the synthesized results.	NA
Reporting bias assessment	14	Describe any methods used to assess risk of bias due to missing results in a synthesis (arising from reporting biases).	Table 3
Certainty assessment	15	Describe any methods used to assess certainty (or confidence) in the body of evidence for an outcome.	NA

RESULTS			
Study selection	16a	Describe the results of the search and selection process, from the number of records identified in the search to the number of studies included in the review, ideally using a flow diagram.	Line 156–223
	16b	Cite studies that might appear to meet the inclusion criteria, but which were excluded, and explain why they were excluded.	NA
Study characteristics	17	Cite each included study and present its characteristics.	Table 1, Table 2
Risk of bias in studies	18	Present assessments of risk of bias for each included study.	Table 3
Results of individual studies	19	For all outcomes, present, for each study: (a) summary statistics for each group (where appropriate) and (b) an effect estimate and its precision (e.g. confidence/credible interval), ideally using structured tables or plots.	Table 1, Table 2
Results of syntheses	20a	For each synthesis, briefly summarise the characteristics and risk of bias among contributing studies.	NA
	20b	Present results of all statistical syntheses conducted. If *meta*-analysis was done, present for each the summary estimate and its precision (e.g. confidence/credible interval) and measures of statistical heterogeneity. If comparing groups, describe the direction of the effect.	NA
	20c	Present results of all investigations of possible causes of heterogeneity among study results.	NA
	20d	Present results of all sensitivity analyses conducted to assess the robustness of the synthesized results.	NA
Reporting biases	21	Present assessments of risk of bias due to missing results (arising from reporting biases) for each synthesis assessed.	NA
Certainty of evidence	22	Present assessments of certainty (or confidence) in the body of evidence for each outcome assessed.	NA

DISCUSSION			
Discussion	23a	Provide a general interpretation of the results in the context of other evidence.	Line 228–348
	23b	Discuss any limitations of the evidence included in the review.	Line 349–352
	23c	Discuss any limitations of the review processes used.	Line 352–354
	23d	Discuss implications of the results for practice, policy, and future research.	Line 360–370

OTHER INFORMATION			
Registration and protocol	24a	Provide registration information for the review, including register name and registration number, or state that the review was not registered.	NA
	24b	Indicate where the review protocol can be accessed, or state that a protocol was not prepared.	NA
	24c	Describe and explain any amendments to information provided at registration or in the protocol.	NA
Support	25	Describe sources of financial or non-financial support for the review, and the role of the funders or sponsors in the review.	NA
Competing interests	26	Declare any competing interests of review authors.	Declaration forms
Availability of data, code and other materials	27	Report which of the following are publicly available and where they can be found: template data collection forms; data extracted from included studies; data used for all analyses; analytic code; any other materials used in the review.	NA

**Table 5:** PRISMA 2020 Abstract checklist.

Section and Topic	Item #	Checklist item	Reported (Yes/No)
*TITLE*			
Title	1	Identify the report as a systematic review.	Yes

*BACKGROUND*			
Objectives	2	Provide an explicit statement of the main objective(s) or question(s) the review addresses.	Yes

*METHODS*			
Eligibility criteria	3	Specify the inclusion and exclusion criteria for the review.	Yes
Information sources	4	Specify the information sources (e.g. databases, registers) used to identify studies and the date when each was last searched.	Yes
Risk of bias	5	Specify the methods used to assess risk of bias in the included studies.	No
Synthesis of results	6	Specify the methods used to present and synthesise results.	Yes

*RESULTS*			
Included studies	7	Give the total number of included studies and participants and summarise relevant characteristics of studies.	Yes
Synthesis of results	8	Present results for main outcomes, preferably indicating the number of included studies and participants for each. If *meta*-analysis was done, report the summary estimate and confidence/credible interval. If comparing groups, indicate the direction of the effect (i.e. which group is favoured).	Yes

*DISCUSSION*			
Limitations of evidence	9	Provide a brief summary of the limitations of the evidence included in the review (e.g. study risk of bias, inconsistency and imprecision).	Yes
Interpretation	10	Provide a general interpretation of the results and important implications.	Yes

*OTHER*			
Funding	11	Specify the primary source of funding for the review.	Yes
Registration	12	Provide the register name and registration number.	Yes

## 4 Discussion

We collated currently available biomarker data from patients with Takotsubo syndrome as a comparator with AMI. A Takotsubo syndrome-specific biomarker does not exist and such a biomarker would require an unprecedented degree of accuracy (implying rapid or pre-existent release of such a protein), ultra-rapid testing availability in order to obviate coronary angiography on those presenting with ST-elevation. It is important therefore to re-appraise if for diagnostic purposes there is particular value in any such existing biomarker or a combination thereof and at what stage in the diagnostic pathway it could be clinically helpful (for example 12 or 24 h, such as is the case with AMI) – this being relevant to those presenting without ST-elevation on ECG. In the absence of high specificity, any clinical biomarker must continue to be utilised in conjunction with other clinical investigations (ECG, and Cardiac imaging) for an accurate diagnosis. A biomarker in Takotsubo syndrome may help select patients that could be assessed non-invasively by computed tomography coronary angiography provided they are stable and pain free [5]. It also may help in follow-up, identification of candidates for long term therapies and those at increased risk.

### 4.1 Acute biomarkers

Cardiac markers of injury and stretch collectively showed that Troponin isoforms, CK or CK-MB are lower in Takotsubo patients compared with STEMI and of comparable or lower levels than patients with NSTEMI [6]. It would be unjustifiable to delay primary percutaneous diagnostic and interventional pursuits in anyone presenting with ST-elevation ECG, the case is however different for the NSTEMI presenters. Both point of care and 12-hour troponin or 12–24-hour CK/CK-MB assays are usually available before further invasive investigations are instigated, although antiplatelet and anticoagulation therapy is commenced in all. However, NSTEMI’s typically have lesser rise in cardiac injury/necrosis biomarkers, thus almost overlapping with those seen in Takotsubo, therefore they do not accurately differentiate Takotsubo syndrome from AMI [7]. Unlike in AMI (where the acute myocardial biomarker release is by and large proportional with the infarct size and thus prognostically important), the amount of Troponin release in the acute Takotsubo stage is not known to have long-term prognostic implications.

An interesting and consistent observation has been the rather high BNP levels in Takotsubo syndrome compared to patients with AMI. A likely explanation is the acute left ventricular ballooning and raised filling pressures in a left ventricular cavity which has suddenly lost its contractile function. BNP is also an acute phase reactant and it is also possible that this elevation reflects its anti-inflammatory contribution to the syndrome [8]. BNP tests are widely available, also as point of care assays [9], [10]. Testing of BNP alone however does not have enough specificity to reliably differentiate Takotsubo syndrome from AMI, particularly in larger infarcts, where BNP can also be very high.

Given the opposite patterns of change in Takotsubo syndrome (lower troponin, higher BNP) compared to AMI, the BNP/troponin ratio appears significantly elevated in patients with Takotsubo syndrome and has a higher sensitivity and specificity for discriminating Takotsubo syndrome from AMI. Doyen et al showed that a ratio greater than 159 had a 95.2% sensitivity and a 97.9% specificity for discriminating patients with Takotsubo syndrome from AMI [6], [11]. Whilst requiring larger studies to validate it for routine use, the benefit of this acute, diagnostic biomarker ratio is that both are already in clinical use.

We have previously demonstrated that Takotsubo syndrome is characterised by intense myocardial and systemic inflammation [2]. Santoro and colleagues evaluated inflammatory markers as diagnostic tools in patients with Takotsubo syndrome. IL-2, IL-4, IL-10, IFN-γ and TNF-α at admission were shown to be significantly higher in patients with acute Takotsubo syndrome compared to patients with AMI, conversely, IL-6 was much higher in AMI patients [12]. This is probably because of the necrotic myocardium in AMI which attracts an immediate and large pool of neutrophil infiltration, which is mainly responsible for the IL-6 rise, whereas necrosis is not characteristically found in Takotsubo myocardium [13]. Consequently, elevated IL-6 levels persist beyond acute presentation [12] in to the subacute phase of AMI [14]. However, the elevated IL-2 and IL-4 levels in Takotsubo syndrome point towards activation of circulating CD4 and CD8 T cells as these are the primary source of these interleukins. The levels of IL-10 which is an anti-inflammatory cytokine rise due to the abundance of macrophages surrounding the myocardial tissue subjected to very high wall stress in Takotsubo syndrome. Thus, there is likely to be more IL-10 released in Takotsubo syndrome compared to AMI patients due to the pro-inflammatory nature of Takotsubo syndrome [12] which needs to be counteracted as part of the mechanism of homeostasis. In addition, IL-10 likely prevents cardiomyocyte apoptosis via TNF-α, accounting for the functional recovery in patients with Takotsubo syndrome [15]. TNF-α and IFN-γ (both pro-inflammatory cytokines) are significantly elevated in Takotsubo syndrome compared to AMI, also likely reflecting the inflammatory substrate of Takotsubo syndrome. TNF-α has direct cardiotoxic effects with reductions in cardiac inotropy; once levels of TNF-α fall this effect is immediately reversible without any residual left ventricular impairment in rats exposed to TNF-α infusions [16]. The levels of IFN-γ are elevated in both hypertensive and aged models of the rat heart demonstrating that any inflammatory process can lead to an accumulation of CD4 and CD8 cells which are a major source of IFN-γ [17]. This supports the strong inflammatory mechanism/circuit for Takotsubo syndrome as there is a preponderance for significantly elevated proinflammatory cytokines compared to AMI patients.

Beyond acute presentation, some of the chemokines and interleukins appear to remain elevated, suggesting a low-grade, chronic inflammatory state after Takotsubo syndrome [2], which we believe contributes to the chronic heart failure phenotype [18].

Takotsubo is also recognised to affect the vascular stress responses. Thus, co-peptin has showed some promise in patients with Takotsubo syndrome as its levels are normal or marginally elevated compared to more substantial elevations in patients with AMI. Other markers of vascular stress such as glycocalyx levels are significantly elevated in Takotsubo compared to healthy controls reflecting endothelial injury that may lead to increased vascular permeability (ultimately responsible for the intense myocardial oedema seen in patients with Takotsubo syndrome) [19]. This also supports the theory of nitrosative stress in Takotsubo syndrome whereby excessive catecholamine stimulation of the myocardium leads to the development of free radicals which cause direct myocyte inflammation which leads to shedding of the glycocalyx and intense myocardial oedema [20]. The levels of syndecan-1 however are much higher in patients with AMI this likely reflects the fact that the level of injury to the vascular architecture in AMI is several folds greater than that which may occur in Takotsubo syndrome. Syndecan-1 likely reflects the degree of overall vascular injury in MI (as it is not correlated with infarct size) and is involved in the vascular repair process [21].

Takotsubo syndrome patients have elevated endothelin levels suggesting possible microvascular vasoconstriction in response to endothelial dysfunction [22]. This contrasts with the vasoconstricting hormone co-peptin (released centrally from the posterior pituitary gland) which is normal or only marginally elevated in Takotsubo syndrome, compared to more substantial elevations in patients with AMI. This suggests the arginine-vasopressin system is not implicated in Takotsubo syndrome [23], [24], [25] or it may be supressed or even exhausted. The differences between these two vasoconstricting peptides may be explained by the local upregulation and secretion of endothelin by local factors such as stretch, hypoxia, free radicals and cytokines whereas co-peptin is released centrally. IL-6 is implicated in the production of vasopressin which could explain the higher level of co-peptin in AMI compared to the lower levels seen in Takotsubo syndrome as the levels of IL-6 are much lower in Takotsubo syndrome, as discussed already [26], [27].

Patients with Takotsubo syndrome have altered microRNA signalling compared to STEMI patients and normal controls. There was upregulation of miRNA 16 and 26a in Takotsubo syndrome, both of which are upregulated in the brains of patients with depression and anxiety [28], [29]. This supports the concept of a Brain-Heart axis which may underpin at least part of the pathogenesis of patients with Takotsubo syndrome and account for the anxiety/depression co-morbidities associated with the condition. The levels of miRNA 1 and 133 which exist in a cluster with each other in the myocardium are upregulated in patients with AMI compared Takotsubo syndrome likely reflecting their role as a marker of myocardial injury and necrosis [30].

### 4.2 Chronic biomarkers

After the acute presentation, in the chronic Takotsubo phase, biomarkers that can provide prognostic information or allow clinical monitoring in the aftermath of Takotsubo syndrome are needed to target emerging therapies and design appropriate surveillance strategies. Several biomarkers have emerged as chronic biomarkers candidates. Scally et al showed that levels of TnI, BNP, IL-6 and IL-8 remain modestly elevated at follow-up in patients with Takotsubo syndrome [2]. BNP was also noticed to be elevated at follow-up by Nguyen et al. [8]. This suggests that the abnormalities in patients with Takotsubo syndrome persist for many months after the acute diagnosis. Detectable levels of troponin and elevated levels of BNP are associated with poorer prognosis in patients with cardiac disease [31], [32]. There is a lack of data on chronic (prognostic) biomarkers and their implication in Takotsubo syndrome.

### 4.3 Limitations

One of the limitations of this systematic review is the large numbers of studies which are retrospective in nature. This increases the likelihood of selection bias and information bias in these studies. This systematic review was performed in as thorough a manner as possible however it is possible a very small number of studies could have been missed by our search strategy.

There has been a rise in the incidence of Takotsubo syndrome of about 4–5-fold during the COVID pandemic [33]. In addition, case-series have shown a significant mortality rate in patients with Takotsubo syndrome and concomitant COVID-19 [34]. This reinforces the urgency for a renewed focus on research in this serious cardiac condition

## 5 Conclusion

Takotsubo syndrome is a serious cardiac condition with an increased incidence in the last decade due to better diagnostic awareness and focused research. There is a diagnostic role for acute biomarkers as a component of the comprehensive criteria for the diagnosis of Takotsubo syndrome, which remains a diagnosis of exclusion. A Takotsubo-specific acute diagnostic biomarker has not yet been described. Biomarkers do help further our understanding of the pathophysiology of Takotsubo syndrome and support the very strong inflammatory substrate to the disease process. There may be a prognostic role for biomarkers in selecting patients who are at higher risk for more aggressive medical therapy in the long-term but there remains a large knowledge gap in this area. There is a need for further biomarker research to address such questions which remain unanswered.

## Author contributions

HK performed and designed the search and drafted the manuscript, DG, AM, HA and AR contributed to the manuscript writing and DD contributed the idea and guided the manuscript writing.

## Declaration of Competing Interest

The authors report no relationships that could be construed as a conflict of interest.

## Appendix A Abbreviations

AMI = Acute Myocardial Infarction
TTS = Takotsubo syndrome
NSTEMI = non-ST-elevation myocardial infarction
STEMI = ST-elevation myocardial infarction
BNP = brain natriuretic peptide
NT-proBNPN-terminal pro B-type natriuretic peptide
GDF-15 = Growth differentiation factor-15
CPK = Creatine Phosphokinase
ACE = Angiotensin converting enzyme inhibitors
IL = Interleukin
TNF = Tissue necrosis factor
IFN = Interferon
EGF = Endothelial growth factor
miRNA = Micro Ribonucleic acid
